# Vinculins interaction with talin is essential for mammary epithelial differentiation

**DOI:** 10.1038/s41598-019-54784-w

**Published:** 2019-12-05

**Authors:** Pengbo Wang, Jian Wu, Amber Wood, Matthew Jones, Robert Pedley, Weiping Li, Robert S. Ross, Christoph Ballestrem, Andrew P. Gilmore, Charles H. Streuli

**Affiliations:** 1 Wellcome Centre for Cell-Matrix Research, FBMH, University of Manchester, Manchester, UK; 2 UCSD School of Medicine, Department of Medicine, La Jolla, CA UK; 3 Veterans Administration Healthcare San Diego, San Diego, CA USA; 4 Present Address: CRUK Manchester Institute, Manchester, UK

**Keywords:** Focal adhesion, Integrins, Differentiation

## Abstract

Vinculin is an essential component of cell adhesion complexes, where it regulates the strength and stability of adhesions. Whilst the role of vinculin in cell motility is well established, it remains unclear how vinculin contributes to other aspects of tissue function. Here we examine the role of vinculin in mammary epithelial cell phenotype. In these cells, correct adhesion to the extracellular matrix is essential for both the formation of polarised secretory acini and for the transcription of tissue-specific milk protein genes. We show that vinculin, through its interaction with talin, controls milk protein gene expression. However, vinculin is not required for the formation of polarised acini. This work reveals new roles for vinculin that are central to cellular differentiation, and for the ability of cells to interpret their extracellular microenvironment.

## Introduction

Animal cells constantly interpret their extracellular environment through the interaction between integrins and the extracellular matrix (ECM)^1^. Integrin-containing adhesion complexes control cell behaviours in metazoans, sensing biochemical and mechanical signals within the microenvironment, thereby allowing cells to form functioning tissues^2^. How adhesion complexes convert this extracellular information into complex and varied intracellular responses is still unclear. A central question that remains unresolved is how specific components of the adhesion complex control physiologically relevant differentiation endpoints.

One of the key proteins involved in sensing mechanical information in adhesion complexes is vinculin^3^. Vinculin is essential for mammalian development, and cells lacking it show diminished adhesion and faster migration^4–7^. Structurally, vinculin consists of a large N-terminal head made up of four separate α-helical bundles (domains D1-D4), a short proline-rich flexible linker, and a C-terminal tail (termed D5)^8^. The head and tail regions interact to form an auto-inhibited inactive conformation, which upon activation opens to allow vinculin recruitment to adhesion complexes^9,10^. In its open conformation, vinculin binds other adhesion complex proteins^11^. The vinculin D1 region binds to talin whilst D5 interacts with F-actin. Thus, in cooperation with talin, vinculin links integrins to the actin cytoskeleton^12^, acting as a force-sensing clutch in cell-ECM adhesions^13^.

Most studies involving vinculin provide mechanistic insights into how it regulates cell-matrix adhesion in 2-dimensional culture models, but it is unclear how vinculin influences tissue-specific function in a 3-dimensional (3D) environment similar to *in vivo* tissue. The ability of mammary epithelial cells (MEC) to form 3D acini that differentiate and express tissue-specific genes provides an ideal opportunity for such structure-function analysis. Acini require coordination of cell-ECM interactions and endocrine signals from prolactin in order to express milk proteins^14,15^. Deletion of β1-integrin inhibited transcription of milk protein genes in mammary gland secretory acini, both *in vivo* and in culture^16^. Furthermore, deletion of β1 integrin prevented MEC from forming polarised 3D acini^17^.

Whilst studies showed that integrins are critical for MEC acini formation, it is not clear how the cytoplasmic components of the integrin adhesion complex coordinate the differentiation and polarisation of MEC in 3D. Here we show that vinculin has surprisingly little impact on acini formation but does have a critical role in MEC differentiation leading to milk production.

## Results

### Vinculin is required for MEC differentiation in 3D

**Figure 1:**
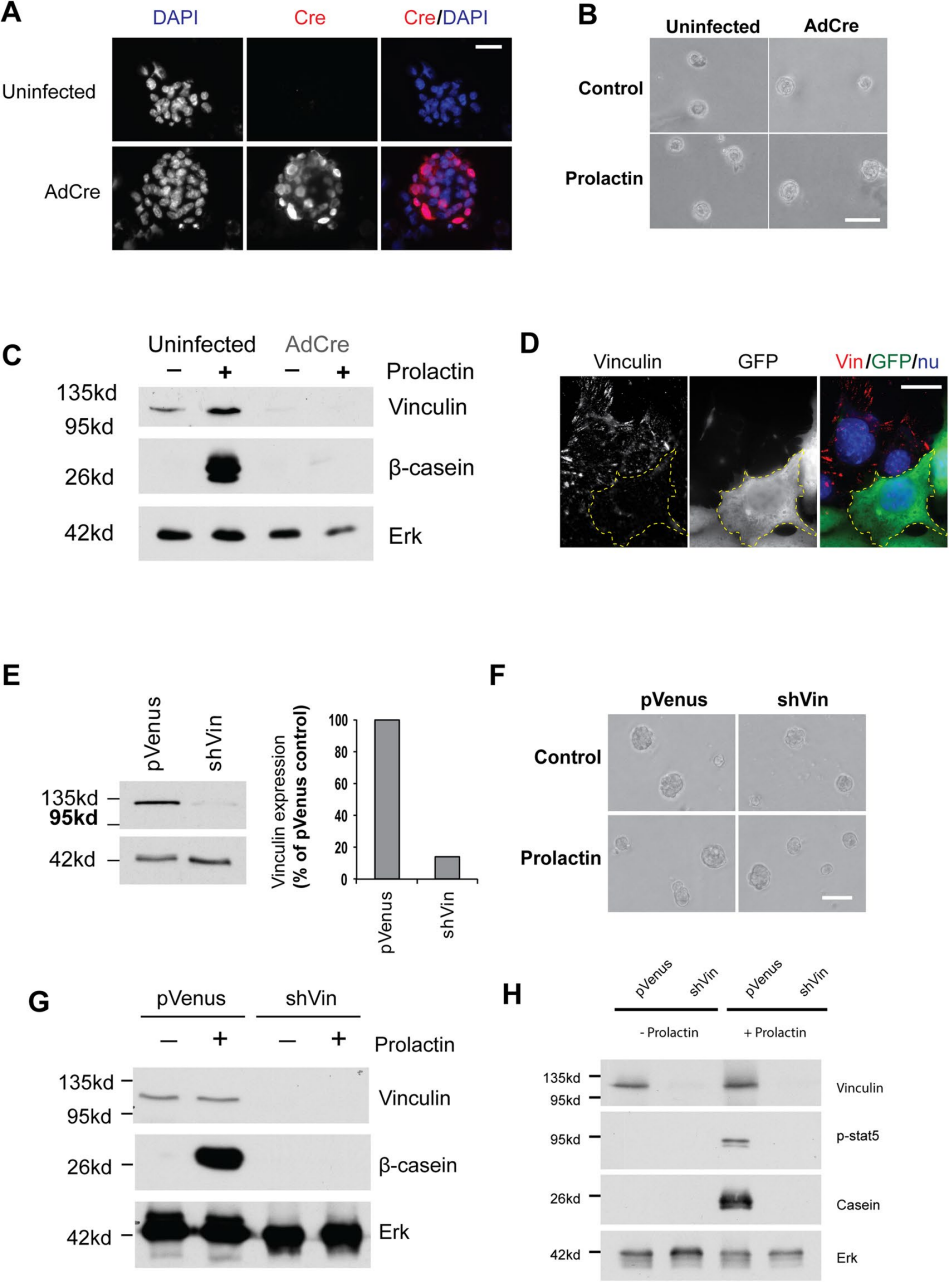
Vinculin expression is required for mammary cells to express milk proteins in 3D-cultures. (**A**) Primary MECs were isolated from 17-day pregnant vinculin^fl/fl^ mice and cultured on collagen-coated dishes for 2 days. Cre-mediated depletion of vinculin was achieved by trypsinising, then incubating single cells with adenovirus expressing Cre recombinase (AdCre) in suspension for 1 hour at 37 °C. Subsequently either untreated or infected cells were cultured in 3D-matrigel for 48 hours. Cells were immunostained for Cre and DAPI, indicating that almost all of the MECs were infected with AdCre. Bar = 50 μm. (**B**) Low-power phase-contrast images showing that both the AdCre infected and control primary vinculin^fl/fl^ MECs formed identical acini in Matrigel, and were not affected by the addition of prolactin. Bar = 100 μm. (**C**) Lysates from the cells in B. were immunoblotted for vinculin and β-casein. Total Erk was used as a loading control. (**D**) Mammary epithelial Eph4 cells were infected with pVenus-shVin and immunostained for vinculin and Venus in 2D. Venus-positive cells did not have detectable vinculin expression. Bar = 20 μm. (**E**) Venus-positive Eph4 cells from D. were sorted by FACS and analysed by immunoblotting for vinculin and Erk. On the right side, quantitative analysis of the data indicated that vinculin expression was reduced by approximately 90% in pVenus-shVin cells, compared with cells infected with pVenus. (**F**) Mock infected Eph4 cells or those infected with shVin-mir were grown in Matrigel for 48 hours, and then treated with prolactin. Phase contrast images (left panel) show no difference in acinar appearance. Bar: 100 μm. (**G**) pVenus and shVin infected Eph4 cells were cultured in Matrigel for 48 hours, and then treated with prolactin. Acini were analysed by immunoblotting for vinculin, β-casein and Erk. (**H**) pVenus and shVin infected Eph4 cells were cultured in Matrigel for 48 hours, and then treated with prolactin. Acini were analysed by immunoblotting for vinculin, p-Stat5, β-casein and Erk. Full-length blots are shown in Supplementary Fig. S4.

To determine how the linkage between integrins and the cytoskeleton coordinates MEC differentiation, we isolated primary cells from vinculin^*fl/fl*^ mice and deleted the vinculin gene with adenoviral (Ad)-Cre recombinase (Fig. 1A). Following vinculin deletion, cells were cultured in 3D-matrigel and compared with control cells. Despite a complete loss of vinculin expression, Ad-Cre-infected vinculin^*fl/fl*^ MECs form similar size acinar structures in 3D-Matrigel as the uninfected cells (Fig. 1B). This result was in marked contrast to when either β1 integrin or ILK are deleted, which leads to the loss of acinar integrity^16,18^. However, despite forming normal sized acini, MECs lacking vinculin failed to differentiate and express β-casein when stimulated with prolactin (Fig. 1C).

To verify this finding, we used Eph4 cells, a mouse MEC line which forms polarised acini in 3D and can be induced to differentiate and secrete milk proteins by addition of prolactin^19^. Eph4 cells were infected with a lentivirus, pVenus, expressing a vinculin targeting shRNA (hereafter termed shVin). Immunofluorescence imaging of shVin-expressing Eph4 cells cultured in 2D showed that they did not have detectable vinculin in adhesions (Fig. 1D). We selected stably-infected shVin Eph4 cells by FACS and analysed vinculin expression by quantitative immunoblotting (Fig. 1E). This indicated ~90% knockdown of vinculin expression in the shVin Eph4 cells compared to pVenus control cells. As with the AdCre infected vinculin^*fl/fl*^ cells, shVin Eph4 cells cultured in 3D Matrigel formed acini that are indistinguishable by phase contrast microscopy to those formed by cells infected with control pVenus (Fig. 1F). In agreement with the data from vinculin^*fl/fl*^ cells, knockdown of vinculin abolished the ability of shVin Eph4 cells to express β-casein in response to prolactin (Fig. 1G). To independently verify the effect of vinculin knockdown on milk protein gene expression, we used the lentivirus pGIPZ to stably express a vinculin-targeted sh-mir (shVin-mir) in Eph4 cells. These cells formed similar sized acini, showed robust vinculin knockdown, but did not express β-casein in response to prolactin (Fig. S1A,B).

Prolactin regulates β-casein gene expression *via* the phosphorylation of the transcription factor Stat5. Our previous studies found that deletion of β1 integrin in MEC inhibits the ability of prolactin to activate Stat5^16^. We therefore asked if the effect of vinculin loss on β-casein expression was due to inhibition of Stat5 activation. We determined whether or not shVin Eph4 cells show phosphorylation of Stat5 in response to prolactin stimulation. In the absence of vinculin, prolactin was unable to activate Stat5, seen by the lack of phosphorylation on tyrosine 694 (Fig. 1H). In contrast, control pVenus-infected Eph4 cells had clear phosphorylation of Stat5 in response to prolactin.

These results show that, as previously seen with β1 integrin, vinculin is necessary for prolactin to activate downstream signalling *via* Stat5, and thereby to permit β-casein gene expression and MEC differentiation.

### Loss of vinculin does not inhibit MEC adhesion complex assembly or acinar polarity

The requirement of vinculin for the prolactin-mediated expression of milk proteins is similar to the necessity of either β1 integrin or ILK^16,17^. However, deletion of the genes encoding the latter proteins had a detrimental effect on the organisation of cell-ECM adhesion complexes in MEC, and resulted in an inability to form polarised acini. We therefore asked whether vinculin deletion or knockdown also resulted in any changes in the ECM-adhesion complexes.

**Figure 2:**
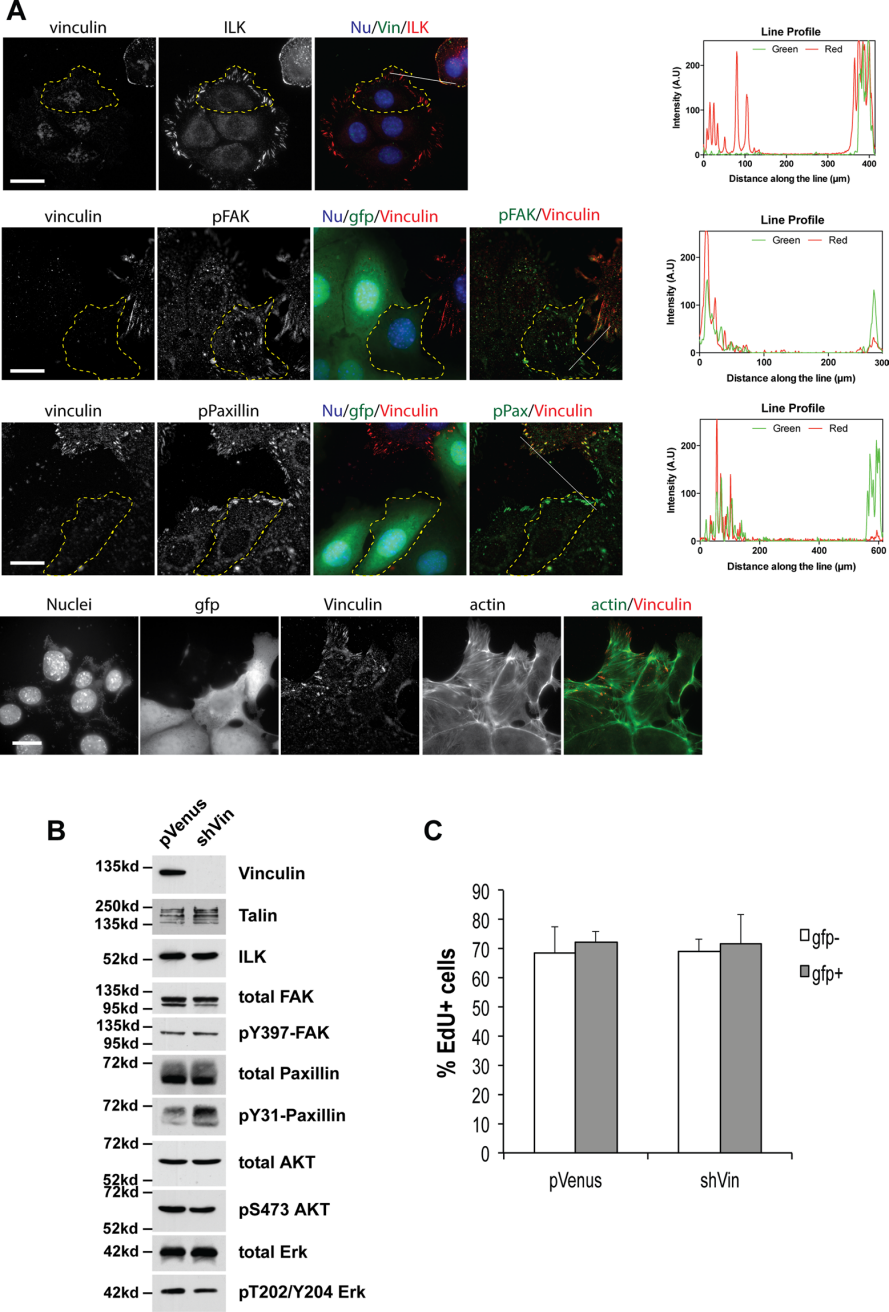
Loss of vinculin expression does not affect cell-ECM adhesions in 2D mammary cell cultures. (**A**) Eph4 cells infected with pVenus-shVin were cultured in 2D and immunostained for vinculin, integrin linked kinase (ILK) phopsho-Y397 FAK, phospho-Y31 paxillin and actin to show similar focal adhesions. Immunostaining with anti-GFP indicates the pVenus–shVin infected cells. Line plots are shown to indicate co-localisation and to compare infected and uninfected cells in the same image. Bars: 20 μm. (**B**) pVenus and pVenus-shVin cells were isolated by FACS. Cells were grown in 2D-culture and lysates probed by immunoblotting for the indicated proteins. No significant changes in any of the focal adhesion proteins, or the phosphorylation of FAK, Erk, or Akt were seen. Full-length blots are shown in Supplementary Fig. S5. (**C**) Eph4 cells infected with pVenus or pVenus-shVin were labelled with EdU before fixing and immunostaining for GFP and EdU. The proportion of EdU-positive cells was quantified. There was no effect of vinculin knockdown on the number of EdU-positive cells, in three independent experiments. Error bars represent SEM.

We first examined the localisation of ILK, phospho-Y397 FAK and phospho-Y31 paxillin by immunofluorescence in Eph4 cells grown in 2D (Fig. 2A). Despite the absence of vinculin, immunostaining revealed the presence of prominent focal adhesions in shVin-Eph4. Apart from the absence of vinculin, the focal adhesions of shVin cells were indistinguishable from uninfected neighbouring cells. To examine potential changes in expression levels of focal adhesion proteins following the loss of vinculin, stable populations of shVin and pVenus control Eph4 cells were isolated by FACS and grown in 2D. Cell lysates from these cells were immunoblotted for a panel of core focal adhesion and signalling components (Fig. 2B). We found that loss of vinculin had no effect on expression levels of talin, ILK, FAK, paxillin, Akt or Erk. Furthermore, there was no loss of phosphorylation of FAK, paxillin, AKT and Erk in the shVin cells relative to the pVenus control cells. To determine whether vinculin loss alters MEC proliferation, Eph4 cells were infected with either pVenus or shVin, grown in 2D and labelled with EdU (Fig. 2C). EdU incorporation was quantified in both the GFP positive (infected) and GFP negative (uninfected) cells. There were no differences in proliferation between uninfected, infected, pVenus or shVin MECs.

**Figure 3:**
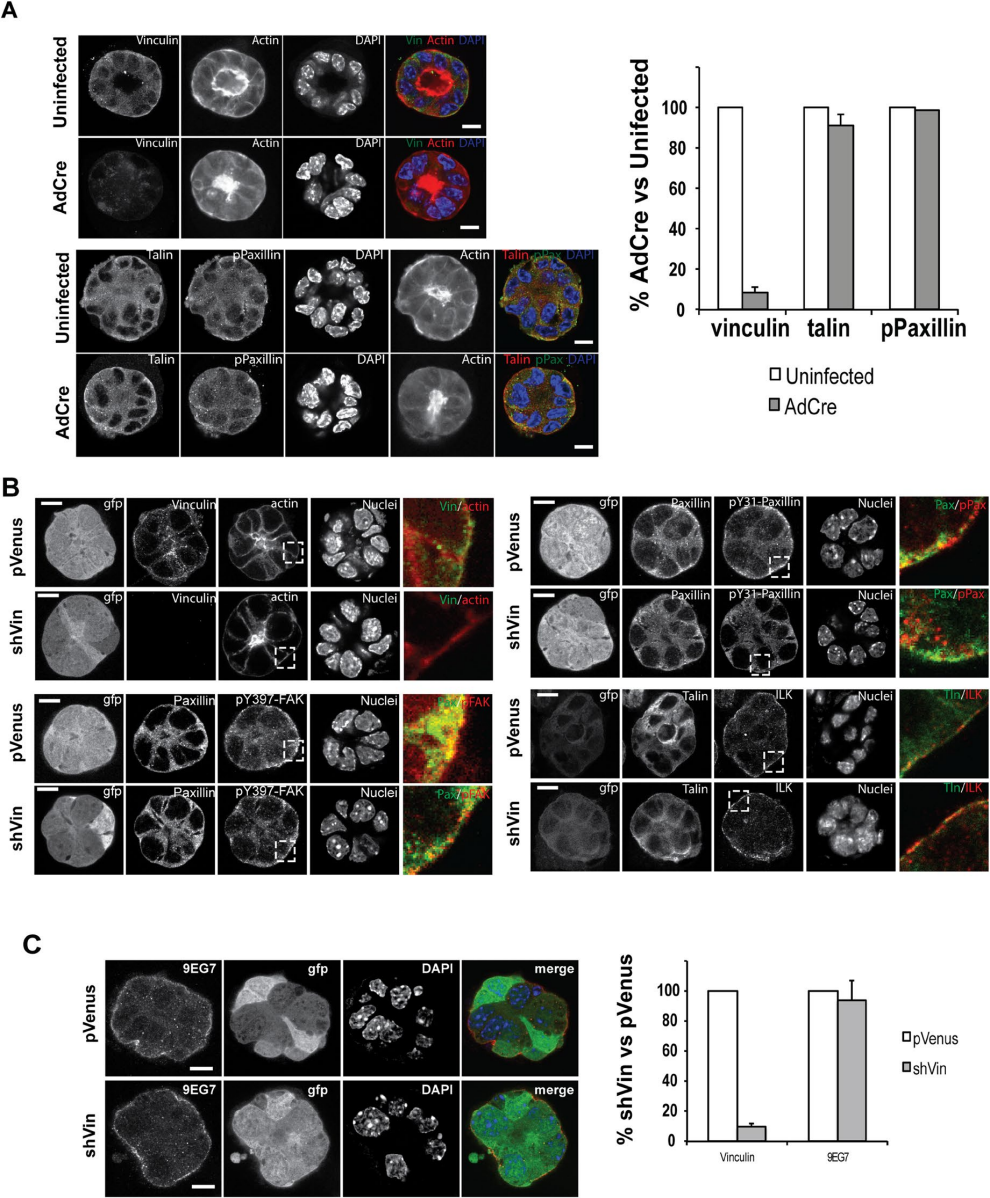
MECs still form polarized acini with correct cell-ECM adhesions in the absence of vinculin. (**A**) Primary MECs isolated from vinculin^fl/fl^ mice were either untreated or infected with adenovirus expressing Cre recombinase (AdCre). The cells were cultured in 3D-matrigel and immunostained for vinculin, talin and phosphor-paxillin. Actin was stained with phalloidin and nuclei with DAPI. Bars: 10 μm. For quantification of vinculin with talin and pPaxillin in both untreated and AdCre treated cells, images were taken using same exposure, the intensity of fluorescence per micrometre squared from region of basal side of spheroid were quantified, and the percentage of intensity of adhesion proteins in AdCre-treated cells compared to uninfected cells. Note the absence of vinculin does not affect the adhesion distribution of talin or pPaxillin. Error bars represent SEM for 3 independent experiments. (**B**) Eph4 cells stably infected with either pVenus or shVin were cultured in Matrigel and immunostained for Venus, vinculin, paxillin, phosphorylated tyrosine 397 focal adhesion kinase (pY397-FAK), phosphorylated tyrosine 31 paxillin (pY31-Paxillin), talin and integrin linked kinase (ILK). Actin was stained with phalloidin and nuclei with DAPI. Bars: 10 μm. (**C**) Eph4 cells infected with pVenus or shVin were grown in matrigel, and active β1 integrin was detected by immunostaining with the monoclonal antibody 9EG7. Bars: 10 μm. Quantification of 9EG7 staining shows that deletion of vinculin doesn’t affect integrin activation, in three independent experiments. Error bars represent SEM.

We next asked whether loss of vinculin impairs the organisation of MEC acini and the formation of adhesion complexes in cells cultured in 3D-matrigel. Vinculin^*fl/fl*^ MEC, either infected with Ad-Cre or uninfected, were cultured in matrigel for 10 days before fixing and immunostaining (Fig. 3A). Despite the loss of vinculin, AdCre-infected MEC formed acini that were indistinguishable from uninfected vinculin^*fl/fl*^ cells, as judged by staining for actin, talin and paxillin. To verify these findings, pVenus- and shVin-infected Eph4 cells were cultured in matrigel for 10 days before immunostaining for adhesion complex components (Fig. 3B). The 3D morphology of vinculin-deficient Eph4 cells was essentially identical to that of control MEC. Furthermore, ILK, paxillin and talin all localised to the basal region of the acini in both control and pVenus cells. Phosphorylation of FAK (pY397-FAK) and paxillin (pY31-paxillin) was apparent in both control and shVin cells, indicating no gross changes in signalling immediately downstream of integrins. Quantification of the basal localisation of talin, paxillin, ILK, paxillin-pY31, FAK-pY397 revealed no differences between pVenus and shVin cells (Fig. S2A,B). As MEC acinar function and milk protein secretion require correct polarisation, we examined the localisation of the tight junction adapter protein, ZO1 (Fig. S2C). Both control and shVin MECs showed an apical/lateral distribution of ZO1, indicating that acini were correctly polarised.

Finally, we determined whether lack of vinculin alters the activation state of β1 integrin on the basal surface of the acini. pVenus and shVin Eph4 cells grown in 3D for 10 days were immunostained with mAb 9EG7 (Fig. 3C). This antibody recognises the activated (ECM bound) conformation of β1-integrin. ECM-bound β1 integrin was detected equally at the basal surface of both vinculin-expressing and vinculin-deficient acini.

These results show that vinculin is not required for either integrin adhesion complex assembly or the formation of acini. Thus, in marked contrast to previous findings for β1 integrin deletion, vinculin deficient MEC have no impairment in their ability to organise into polarised 3D acini.

### The vinculin head domain is sufficient for MEC differentiation

**Figure 4:**
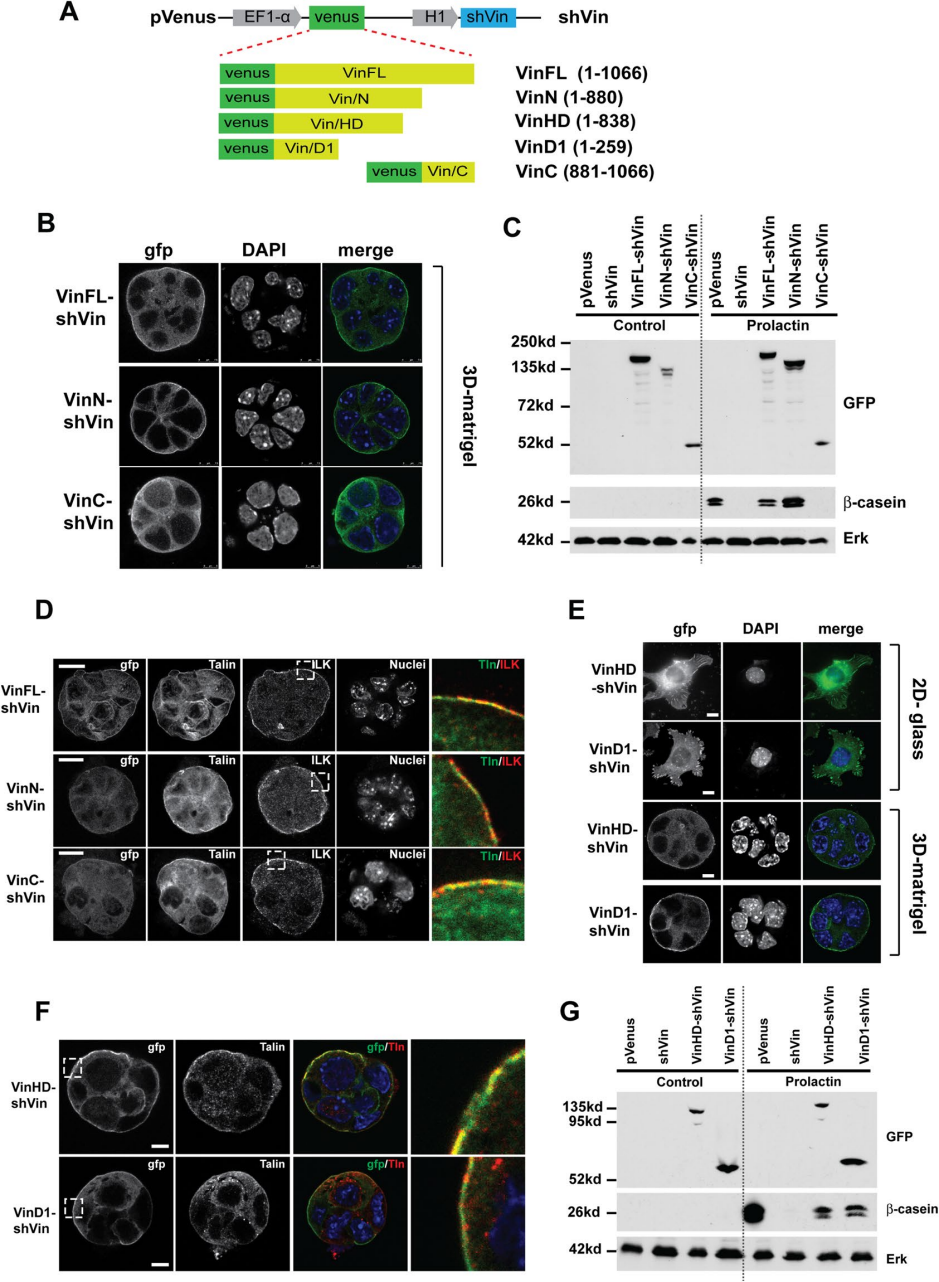
Milk protein expression requires the talin binding D1 domain of vinculin. (**A**) Design of the pVenus vinculin shRNA knockdown constructs. For the rescue experiments, the indicated chicken vinculin sequences were inserted in frame with the pVenus coding sequence. (**B**) GFP-immunostaining of Eph4 cells in 3D-Matrigel infected with the lentiviral constructs VinN-shVin, VinC-shVin or VinFL-shVin indicates that both full-length vinculin and the vinculin N-terminus show strong localization to the basal adhesions, while vinculin C-terminus is more cytoplasmic. Bar: 10 μm. (**C**) Eph4 cells infected with pVenus, shVin, VinFL-shVin, VinN-shVin or VinC-shVin were grown in Matrigel and either left untreated or treated with prolactin for 48 hours. Lysates were immunoblotted for Venus, β-casein or Erk. (**D**) Eph4 cells expressing VinN, VinC or VinFL show no significant alterations in adhesion complexes in 3D matrigel, as seen by immunostaining for talin and ILK. Bars: 10 μm. (**E**) Eph4 cells infected with either VinHD-shVin or VinD1-shVin were grown on 2D coverslips (top 2 panels) or in 3D Matrigel (bottom 2 panels). Cells were immunostained for GFP and nuclei were stained with DAPI. Bar = 10 μm. (**F**) VinHD and VinD1 expression in 3D-acinin did not alter the basal localization of talin, as seen by immunostaining. Bar = 10 μm. (**G**) Eph4 cells infected with pVenus, shVin, VinHD-shVin or VinD1-shVin were grown in Matrigel and either left untreated or treated with prolactin for 48 hours. Lysates were immunoblotted for Venus (GFP), β-casein, vinculin or Erk. Full-length blots are shown in Supplementary Fig. S6.

Vinculin is a multidomain protein with individual regions having the capability of interacting with other adhesion complex proteins^11^. To determine the contribution of vinculin domains for milk expression, we generated pVenus lentiviral vectors which co-expressed both shVin to knockdown endogenous vinculin and either full-length (VinFL) or vinculin deletion constructs. We used chicken vinculin for the rescue constructs, as it was not targeted by shVin. Deletion constructs included the vinculin head and neck region (VinN, residues 1–880), or the actin-binding tail (VinC, residues 881–1066) (Fig. 4A). These lentiviral constructs were stably transduced into Eph4 cells (Fig. S3A,B).

In 2D culture, VinFL, VinN and VinC localised to focal adhesions, although VinC appeared more cytoplasmic (Fig. S3C). When these lines were cultured in 3D, both VinFL and VinN localised strongly at the basal surface of the acini, while VinC was more evenly distributed throughout the cell (Fig. 4B). We asked if any of these vinculin domains rescued the ability of shVin cells to express β-casein in response to prolactin. Both VinFL and VinN, but not VinC, restored β-casein expression of shVin MECs in 3D culture (Fig. 4C). Immunofluorescence showed a normal localisation of talin and ILK, indicating that replacing endogenous vinculin with VinFL, VinC or VinN had no effect on adhesion complex formation in 3D acini (Fig. 4D).

Vinculin interacts with other adhesion complex proteins, including Arp 2/3, VASP and members of the vinexin family, through the flexible proline-rich neck^11^. By further deleting the neck region from VinN to achieve a construct consisting of domains D1-4 (residues 1–838; VinHD; Fig. 4A), we aimed to assess whether any of the neck binding proteins have the potential to mediate the response to prolactin. We also tested a construct comprising the N-terminal amino acids (residues 1–258; VinD1; Fig. 4A), which comprises the minimal region to bind talin (Fig. 4A). VinD1 has previously been shown to activate the integrin/talin complex leading to the stabilisation of focal adhesions in cells cultured in 2D^20^. As expected, both Venus-tagged VinHD and VinD1 localised to the basal surface of acini in 3D Matrigel, and to focal adhesions in cells cultured in 2D on glass (Fig. 4E). The 3D-acini were indistinguishable from wild type cells, with talin co-localising with the Venus-vinculin constructs at the basal surface (cf. Figu. 4F with Fig. 3). When these acini were treated with prolactin, both VinHD and VinD1 rescued β-casein expression in shVin cells (Fig. 4G).

These results show that the ability of vinculin’s head region to bind talin is critical for milk protein expression in 3D culture. Interactions of vinculin with other binding partners that interact with the neck and tail regions are not required.

### Vinculin’s requirement for differentiation is not linked to cell-cell adhesion

Vinculin is also present at cell-cell adhesions, where it can link cadherins to the actin cytoskeleton *via* catenins^21^. Although the Venus-vinculin constructs predominantly localise to ECM-junctions, we could not discount a requirement for cell-cell junctions in controlling milk protein expression. To address this, we used a single cell assay where cadherin-mediated junctions could not be formed.

**Figure 5:**
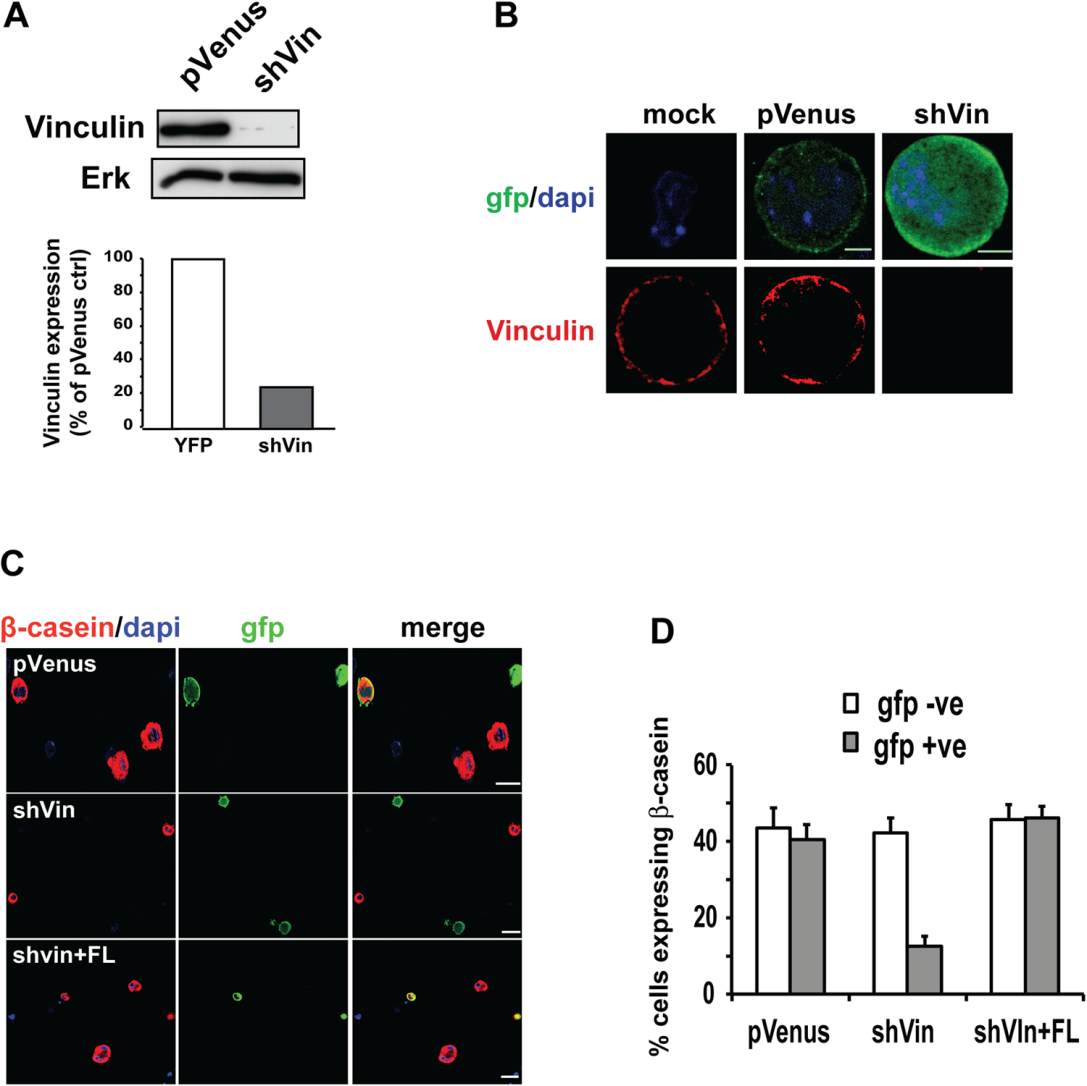
The role of vinculin in regulating milk expression is not dependent upon cell-cell adherens junctions. (**A**) Primary MECs were infected with either pVenus or shVin for 48 hours, then Venus-positive cells were sorted by FACS and analysed by immunoblotting for vinculin and Erk. Quantitative analysis of the data indicated that vinculin expression was reduced by approximately 80% in shVin cells, compared with cells infected with pVenus. (**B**) Primary MECs, infected with either pVenus or shVin, were plated as single cells in Matrigel and immunostained for GFP or vinculin. Immunostaining of individual pVenus cells show localization of vinculin at the cell membrane. shVin cells show no detectable vinculin staining. Bars = 1 μm. (**C**) Single cell cultures of primary MECs in Matrigel were treated with prolactin, before cells were immunostained for Venus and β-casein. Viral-infected cells were identified by immunostaining for Venus. Bar = 5 µm. (**D**) The number of β-casein positive cells in C. was determined for both the infected (GFP +ve) and uninfected (GFP −ve) cells. For each condition, cells were counted in three independent experiments. Bars: 5 μm. Error bars represent SEM.

Primary MEC directly isolated from the mammary glands of mid-pregnant mice, when grown as single cells within 3D-matrigel, still respond to prolactin and make β-casein^15^. To confirm that shVin is able to knock down vinculin expression in primary MECs, we infected cells with either pVenus or shVin, and sorted them by FACS based on GFP expression. Sorted cells were immediately lysed in sample buffer for immunoblotting (Fig. 5A). shVin knocked down vinculin expression in primary MEC to the same extent as in Eph4 cells (cf. Fig. 1E). Infected primary cells were then grown as single cells in 3D, and immunostained for GFP and vinculin (Fig. 5B). Wild-type and control pVenus-infected MEC had endogenous vinculin located at the cell-matrix interface. In contrast, vinculin was absent in shVin-infected cells.

Primary MECs infected and sorted by FACS cannot be grown in culture to obtain sufficient material for immunoblotting milk proteins. However, β-casein expression can be determined in MEC at the single cell level by immunostaining in 3D^15^. We transiently infected MEC with the shVin lentivirus and directly quantified β-casein expression in infected and uninfected cells within the same population. Primary MECs were infected with pVenus, shVin or VinFL-shVin, embedded as single cells in matrigel, then treated with prolactin. Immunostaining was performed to quantify β-casein expression in both the GFP-positive (infected) cells and GFP-negative (uninfected) cells (Fig. 5C). Consistent with previous studies^15^, around 40% of uninfected single MECs stained positively for β-casein, and this was identical in cells infected with pVenus alone (Fig. 5D). For each condition, the level of β-casein expression in the uninfected GFP-negative cells was identical. In contrast, shVin-infected MEC showed a significant reduction in the number of single cells expressing β-casein. β-casein expression was rescued in shVin cells by the expression of full-length chicken vinculin.

These results show that the ability of vinculin to regulate milk protein expression is independent of cell-cell adhesions.

## Discussion

In this study we have identified a novel requirement for vinculin in controlling cell-type specific gene expression. Vinculin is required for milk protein gene expression, but not for MEC to form polarised acini or to organise cell-matrix adhesions in a 3D context. These data indicate that the role of vinculin in sensing the extracellular microenvironment in 3D is crucial for some aspects of cell function, but that it is subtler than predicted from 2D models of focal adhesion regulation.

The mammary gland is an excellent model for deciphering the role of the ECM in supporting the formation and function of 3D tissues^2^. MEC interactions with laminin within the basement membrane, *via* β1-integrins, are necessary for the prolactin receptor to activate Stat5 and drive milk protein gene expression^16^. Several studies have defined the control of prolactin signalling through integrin in adhesion complexes, using *in vivo* genetics and 3D cell culture models^22^. Deletion of either β1-integrin or ILK results in the loss of both 3D organisation and MEC polarity of MEC acini, along with an inability to express milk in response to prolactin^17,18^. Whereas Rac1 can restore milk protein gene expression in ILK-deficient MEC, Rac1 itself is not required for the formation of polarised acini^23^. Thus, integrins appear to initiate multiple signals to coordinate both acinar morphogenesis and MEC-specific gene expression. The results presented in this study now indicate that vinculin plays a previously unknown role in coordinating tissue-specific gene expression. It is required for prolactin to activate Stat5, but it is not necessary for 3D cellular organisation.

Vinculin is found in both cell-ECM and cell-cell adhesions, and it is known that force transmission has distinct regulatory mechanisms in these contact sites^24^. We have now discovered that vinculin is only required at cell-ECM adhesions for MEC differentiation. A key role of vinculin in adhesion complexes is the transmission of mechanical force from the acto-myosin cytoskeleton to the ECM^25^. In ECM adhesions, vinculin interacts with both talin and actin through N- and C-terminal binding sites respectively, and this linkage is required for acto-myosin contractility to be transmitted from the ECM^6,20^. Absence of vinculin, whilst not disrupting focal adhesions in 2D, does alter the size and dynamics of adhesion complexes, resulting in altered migration of fibroblasts^6^. In the study presented here, we found no gross change in ECM adhesion complex composition in the absence of vinculin in epithelial cells grown in either 2D or 3D. Interestingly, full-length vinculin was not essential for milk protein gene expression, and differentiation was rescued in vinculin-deficient cells just by the N-terminal talin binding D1 domain. VinD1 lacks the tail region linking it to actin, and therefore it seems that a force transmitting linkage through vinculin is not required for MEC differentiation, although it may have a role in activation of full-length vinculin.

How vinculin functions to link adhesion signalling with prolactin signalling remains unclear. One possibility is that its role is indirect, through stabilisation of other adhesion complex components. In 2D, the talin-binding D1 domain is sufficient to stabilise talin in adhesion complexes, and it can do this independently of acto-myosin contractility^20^. As the talin binding D1 domain of vinculin is sufficient to rescue the expression of milk proteins, we can discount roles for other proteins interacting with vinculin, either in the tail or the neck regions. If talin binding is all that is required for vinculin to allow MEC differentiation, then it is likely that the adhesion complexes in 3D show a degree of dynamic turnover comparable with FAs in 2D. Furthermore, we can speculate that these dynamics are central to how MEC perceive their extrenal microenvironment and respond appropriately through downstream signalling. What the signals are remains to be determined, as we found no gross differences in FAK or paxillin phosphorylation between vinculin-expressing and -deficient MECs, either in 2D or 3D.

In conclusion, our results demonstrate that vinculin has a crucial role in orchestrating the differentiation of epithelia in their normal 3D context. Vinculin does not just have a role in adhesion complex assembly and cell migration, but is critically important for the expression of tissue-specific genes^5,26^. Our data alos reveal that the linkage between vinculin and talin is important for its signalling capacity, and highlights novel functions for these proteins in epithelial cells.

## Methods

Methods are essentially as described in our previous study^27^.

### Cell culture

EPH4 cells were derived from spontaneously immortalized mouse mammary gland epithelial cells. EPH4 cells were cultured in DF12 medium (BioWhittaker; Lonza) supplemented with 2.5 μg/ml insulin, and 5% FCS at 37 °C in a humidified atmosphere of 5% CO_2_. Primary MECs from were extracted from 19-d pregnant vinculin^fl/fl^^28^ or WT mice as previously described^29^, and cultured in growth medium containing 5 μg/ml insulin, 1 μg/ml hydrocortisone, 3 ng/ml EGF, 10% FCS, 50 U/ml penicillin/streptomycin, 0.25 μg/ml fungizone, and 50 μg/ml gentamycin in F12 medium. All the lentivirus constructs were generated in HEK293T cells cultured in DMEM medium supplemented with 10% FCS, as previously described. Note that infecting primary MECs with control viruses, e.g. expressing β-gal or LacZ, has no deleterious effect on their expression of adhesion complex proteins or milk proteins, or on cell shape in 2D or 3D culture (Naylor *et al*.^16^).

### DNA constructs

The lentiviral shRNA vector, pVenus, was provided by Didier Trono (University of Geneva, Geneva, Switzerland). shRNA for mouse vinculin was designed with shRNA design tool (Open Biosystems). The target sequences for mouse vinculin were the following. shVin: 5′-CGAGATCATTCGTGTGTTA-3′; shVin-mir: 5′-CGGATTAGAACCAATCTCTTA-3′. A BLAST search did not reveal any other target sequences in mouse. Doubled-stranded oligonucleotides were cloned into the lentiviral transfer vectors pVenus (shVin) or pGIPZ (shVin-mir).

For the rescue vectors, chicken wild-type vinculin was used to avoid knockdown by the shVin. Chicken and mouse vinculin have almost identical amino acid sequences. Full length (aa1-1066), N’-terminus (aa1-880), C’-terminus (aa881-1066), head domain (aa1-838) and D1 domain (aa1-259) of vinculin were cloned into pVenus. The expression of Venus (a variant of YFP) tagged rescue proteins were regulated by EF1a promoter, with the endogenous vinculin knockdown were achieved by shVin introduced downstream of H1 promoter in the same vector.

### Lentiviral infection

For lentivirus production, the transfer vectors were co-transfected with the envelope plasmid pMD2G and the packaging plasmid psPAX2 into HEK293T cells using PEI reagent. Media were replaced after 8–10 h. 10 ml viral supernatant was harvested 48–60 h after transfection, passed through a 0.45-μm filter, and further concentrated by centrifugation at 25,000 rpm at 4 °C for 2.5 h. Viral pellets were re-suspended in 0.1 ml fresh DF12 medium.

Lentiviral infection was performed by adding lentiviral particles directly to pre-cultured 75% confluent cells and followed by incubation for 3 h. The infected cells were cultured for 48 h to ensure turnover of pre-existing vinculin. Pure populations of infected cells were enriched by FACS sorting the Venus (pVenus) or GFP (pGPIZ) positive cells.

### Differentiation assay

Cells plated onto basement membrane-matrix (Matrigel BD Biosciences) formed 3D acini 24 h after plating. Subsequently cells were cultured in differentiation DF12 media containing 5 μg/ml insulin and 1 μg/ml hydrocortisone for 48 h. 3 μg/ml prolactin (Sigma) was added and incubated for 48 h for the endpoint assays.

For the single cell assay, lentiviral infected primary MECs were trypsinized, passed through a 40 μm filter to remove cell aggregates and the remaining single cells were resuspended in Matrigel. 100 μl cell suspension was pipetted onto a thin monolayer of Matrigel, set at 37 °C, and cultured in growth medium for 24 h, and then differentiation medium containing 5 μg/ml prolactin for a further 48 h. Cells were fixed with 4% formaldehyde in PBS and immunostained. Cells were visualized by immunofluorescence, and β-casein expression was quantified by nonbiased cell counts.

### Immunofluorescence

Cells were fixed with 4% formaldehyde for 10 min, permeabilized for 5 min with 0.5% Triton X-100 (Sigma-Aldrich), and subsequently incubated for 1 h with primary antibodies and 0.5 h with appropriate secondary antibodies (Jackson ImmunoResearch Laboratories, Inc). For actin fluorescence staining, Alexa Fluor 647–phalloidin (Invitrogen) was added to cells together with the secondary antibody.

Antibodies used for immunofluorescence: vinculin and talin (Sigma-Aldrich); 9EG7 and paxillin (BD); ILK and pPaxillin-Y31 (Cell Signalling); pFAK-Y397 and GFP (Invitrogen); all secondary antibodies (Jackson ImmunoResearch Laboratories, Inc.). After staining, coverslips were mounted with antifade reagent (Pro-Long; Invitrogen). Mouse anti–β-casein was described previously^15^.

Mouse anti-ILK was a gift from C.Wu. Commercial primary antibodies were as follows: vinculin, talin (Sigma); β1-integrin^30^; 9EG7 (BD Pharmingen); paxillin (BD transduction labs); phospho-paxillin Y118 and phospho-FAK Y397 (Biosource International); V5 and GFP (Invitrogen); Stat5a, Erk (Santa Cruz Biotechnology, Inc.). Secondary antibodies were anti-mouse, -rabbit, and –rat cy2 or Rhrx or cy5 (Jackson ImmunoResearch Laboratories).

Images of 3D-acinar structure were captured using a Leica TCS SP5 AOBS inverted confocal microscope using a ×63 Plan Fluotar objective. Images of 2D-cultured cells were acquired on an Olympus microscope equipped with a Deltavision imaging system using either a 100x/1.40 Uplan S Apochromator a 60x/1.42 Plan Apochromat objective. Delta vision images were processed by constrained iterative deconvolution using SoftWoRx (Applied Precision). All images analysis was performed using ImageJ.

### Immunoblotting

For immunoblotting, proteins were extracted from cell lysates using NET buffer (50 mM Tris-Cl pH 7.6, 150 mM NaCl, 1% NP-40, 2 mM EDTA, 1 mM sodium orthovanadate, 10 mM sodium fluoride) supplemented with 1:100 dilution of Protease cocktail inhibitor set 1 (Calbiochem). Equal amounts of proteins were separated by SDS -PAGE and immunoblotted for proteins. Specific binding was detected with HRP-conjugated secondary antibodies (Jackson laboratories), followed by chemiluminescence (Amersham Biosciences). For quantitative analysis of vinculin knockdown, IR labelled secondary antibodies were used (Jackson) in conjunction with a LiCor Odyssey imaging system.

## Supplementary information

Supplementary data
