# Supplementary material for: Vinculins interaction with talin is essential for mammary epithelial differentiation
Source: Sci Rep. 2019 Dec 5;9:18400. doi: 10.1038/s41598-019-54784-w (PMC6895056; doi:10.1038/s41598-019-54784-w)
Supplement: Supplementary file 1 — Supplementary data [file 41598_2019_54784_MOESM1_ESM.pdf]

Vinculins interaction with talin is essential for mammary epithelial differentiation.

Pengbo Wang[1,3], Jian Wu[1], Amber Wood[1], Matthew Jones[1], Robert Pedley[1], Weiping Li[1], Robert S. Ross[2], Christoph Ballestrem[1¤], Andrew P. Gilmore[1¤*] and Charles H. Streuli[1¤].

[1]Wellcome Centre for Cell-Matrix Research, FBMH, University of Manchester, Manchester, UK.

[2]UCSD School of Medicine, Department of Medicine, La Jolla, CA, and Veterans Administration Healthcare San Diego, CA, USA

[3]Currently at: CRUK Manchester Institute, Manchester, UK

¤ Joint senior authors; * corresponding author

Supplementary data and legends

Supplementary data

Figure S1. Vinculin depletion does not alter acinar morphology.

A. Design of the shVin-mir lentivirus.

B. Mock infected Eph4 cells or those infected with shVin-mir were grown in Matrigel for 48 hours, and then treated with prolactin. Phase contrast images (left panel) show no difference in acinar appearance. Bar = 100 μm.

C. WT Eph4 acini or those infected with shVin-mir from B. were analysed by immunoblotting for vinculin, β-casein and Erk.

Figure S2. MECs still form polarized acini with correct cell-ECM adhesions in the absence of vinculin.

A. Quantification of the data in Figure 3. The areas between the double yellow dotted lines show the regions that were examined for quantification of staining. B. Quantification from A. of talin, paxillin, ILK, pPaxillin and pFAK in the presence and absence of vinculin. Note that deletion of vinculin by shVin did not alter the basal location of these proteins in Matrigel-cultured acini, in three independent experiments. Error bars represent SEM.

C. Eph4 cells, uninfected controls or cells infected with either shVin or FL-vinculin and shVin, were grown in Matrigel, and immunostained for vinculin and ZO1. GFP-was visualised directly. Bar = 10 μm

Figure S3. Expression of vinculin rescue constructs in Eph4 cells using pVenus.

A. Confirmation of knockdown of endogenous vinculin and expression of the vinculin (amino acids 1-880). As VinN was the same molecular weight as endogenous vinculin, it was not possible to confirm complete knockdown with this construct using simple immunoblotting. We therefore passed lysates over

GFP-trap agarose beads to extract the expressed Venus-VinN, whilst any endogenous vinculin passed through. Immunoblotting of the beads and the flow through indicate that in the case of empty pVenus (with no vinculin sh sequence), endogenous vinculin was detected in the flow through at the same level as in the input lysate. In contrast, cells infected with pVenus VinN-shVin show no endogenous vinculin in the flow through, and extraction of the expressed VinN on the beads.

B. Expression of Venus-tagged VinC was detected with an anti-GFP antibody, since VinC doesn’t contain the epitope for the vinculin antibody.

C. Eph4 cells infected with the lentiviral constructs VinN-shVin, VinC-shVin or VinFL-shVin, were grown in 2D on coverslips and immunostained for Venus (GFP) and paxillin, Bar: 20 μm.

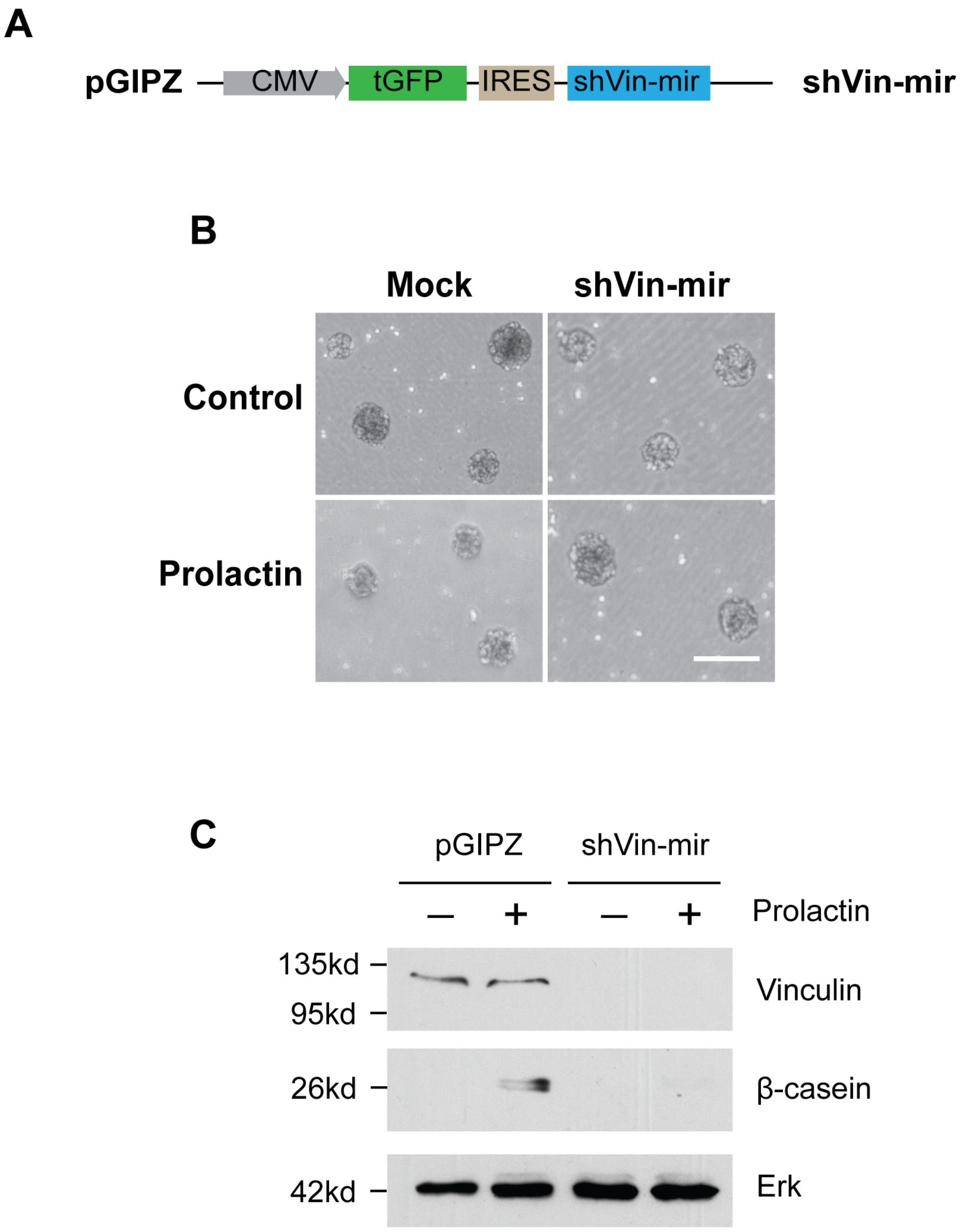

Wang et al. Figure S1

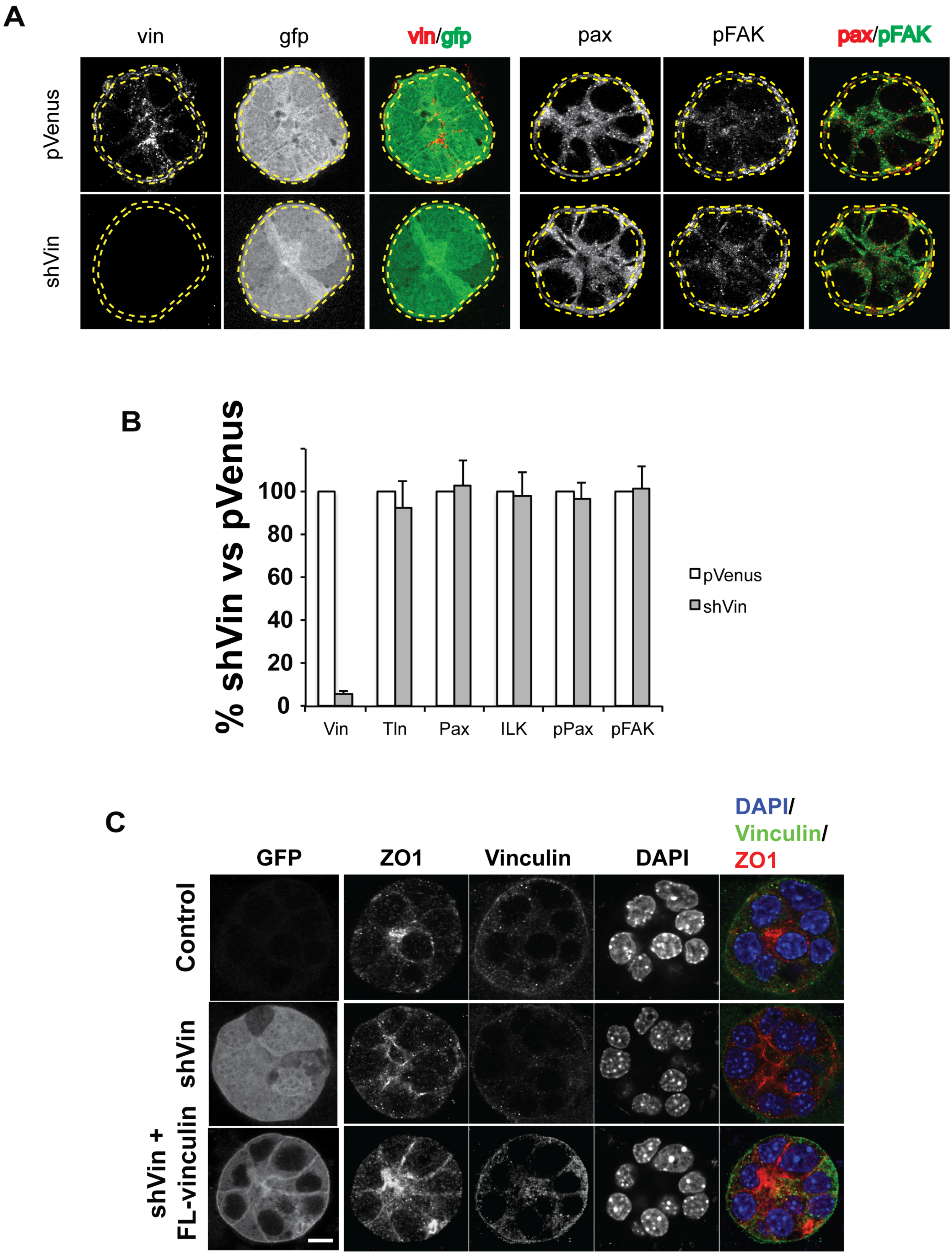

Wang et al. Figure S2

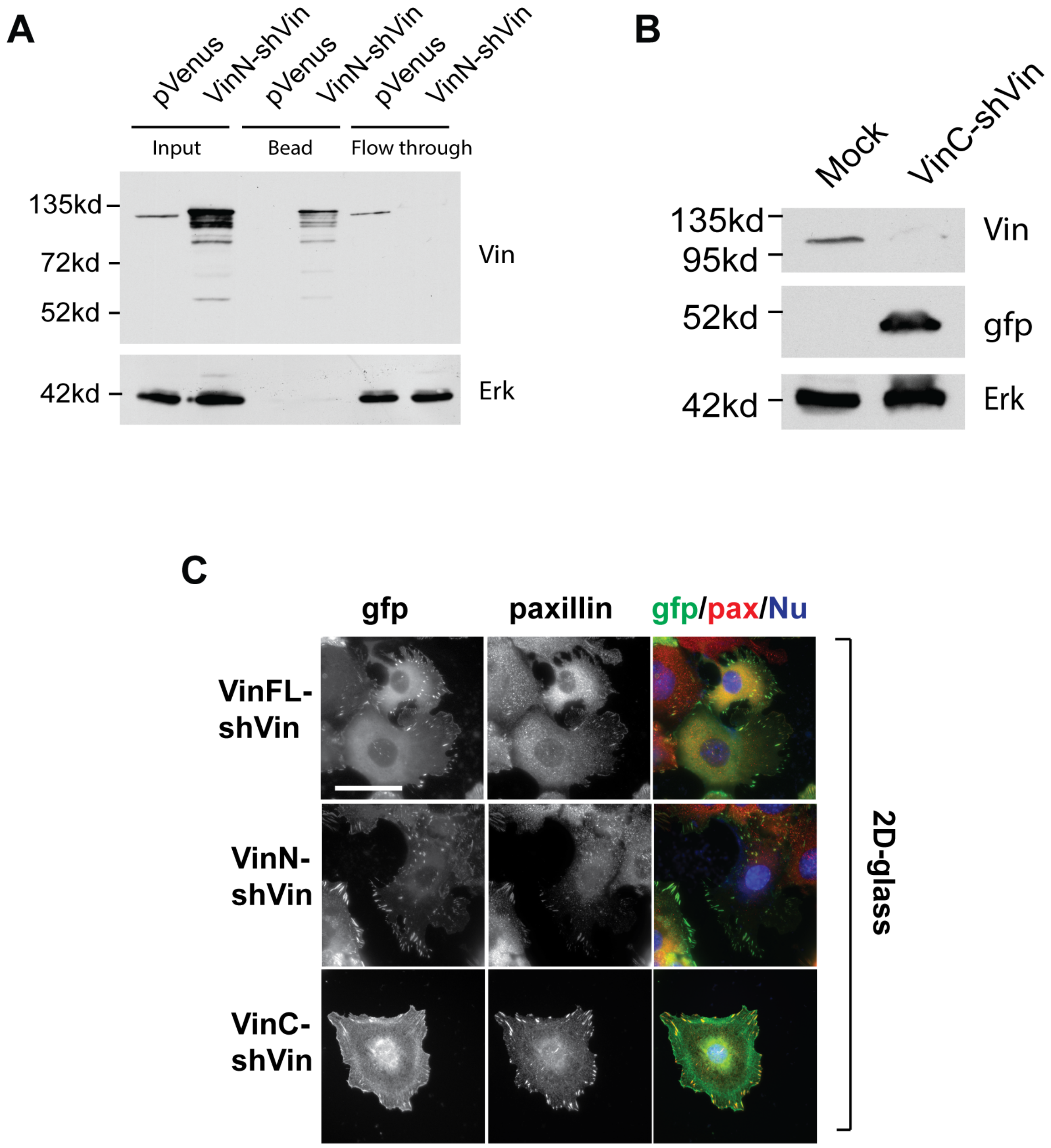

Wang et al. Figure S3

Figure 1C

long exposure

short exposure

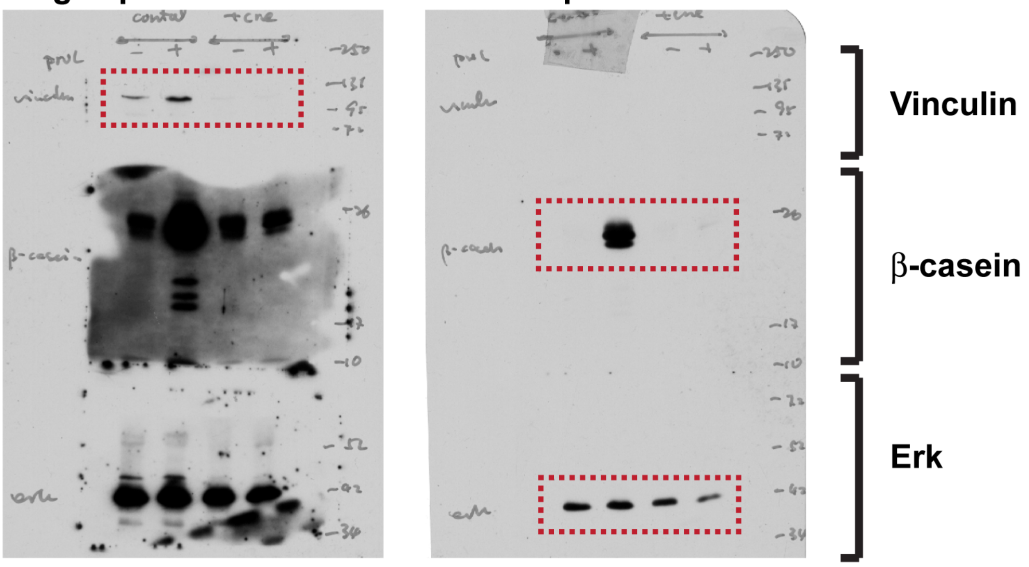

Figure 1E

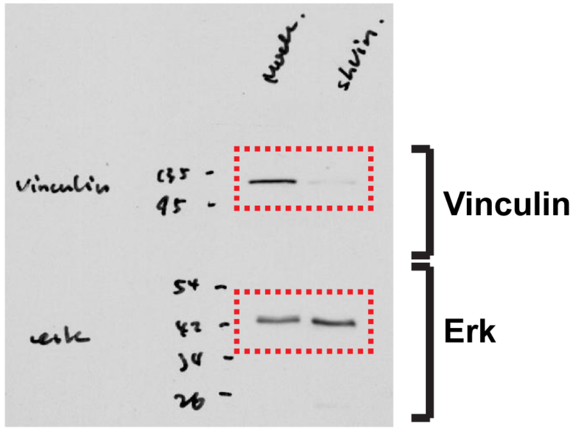

Figure 1G

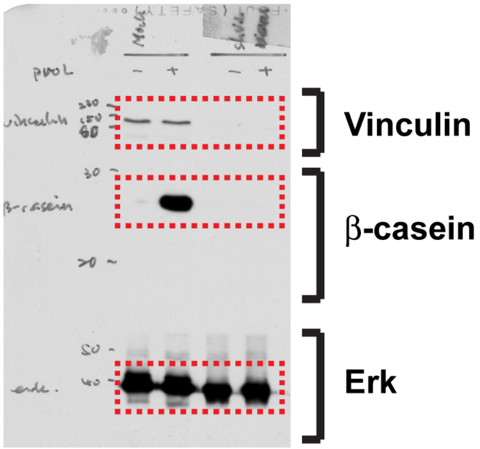

Figure 1H

long exposure

short exposure

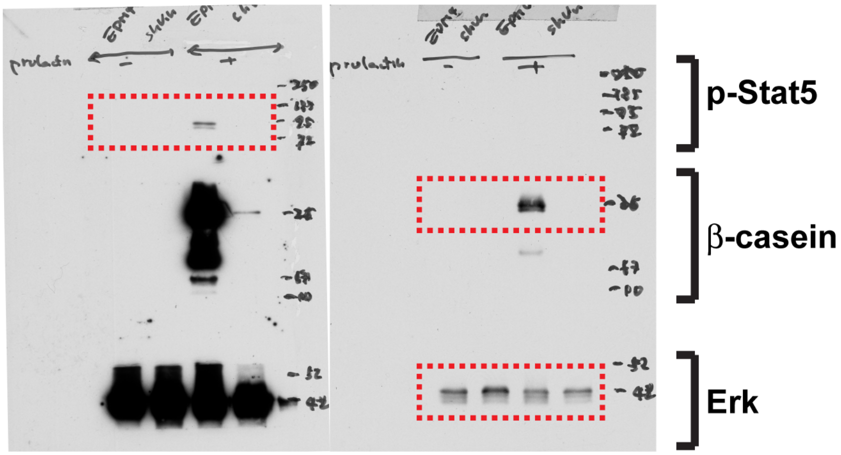

Figure 1H vinculin

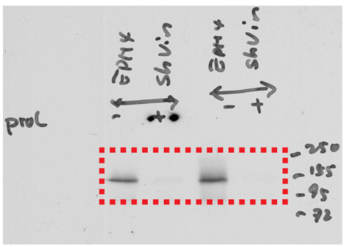

Uncropped blots Figure 1

Wang et al. Figure S4

Figure 2B

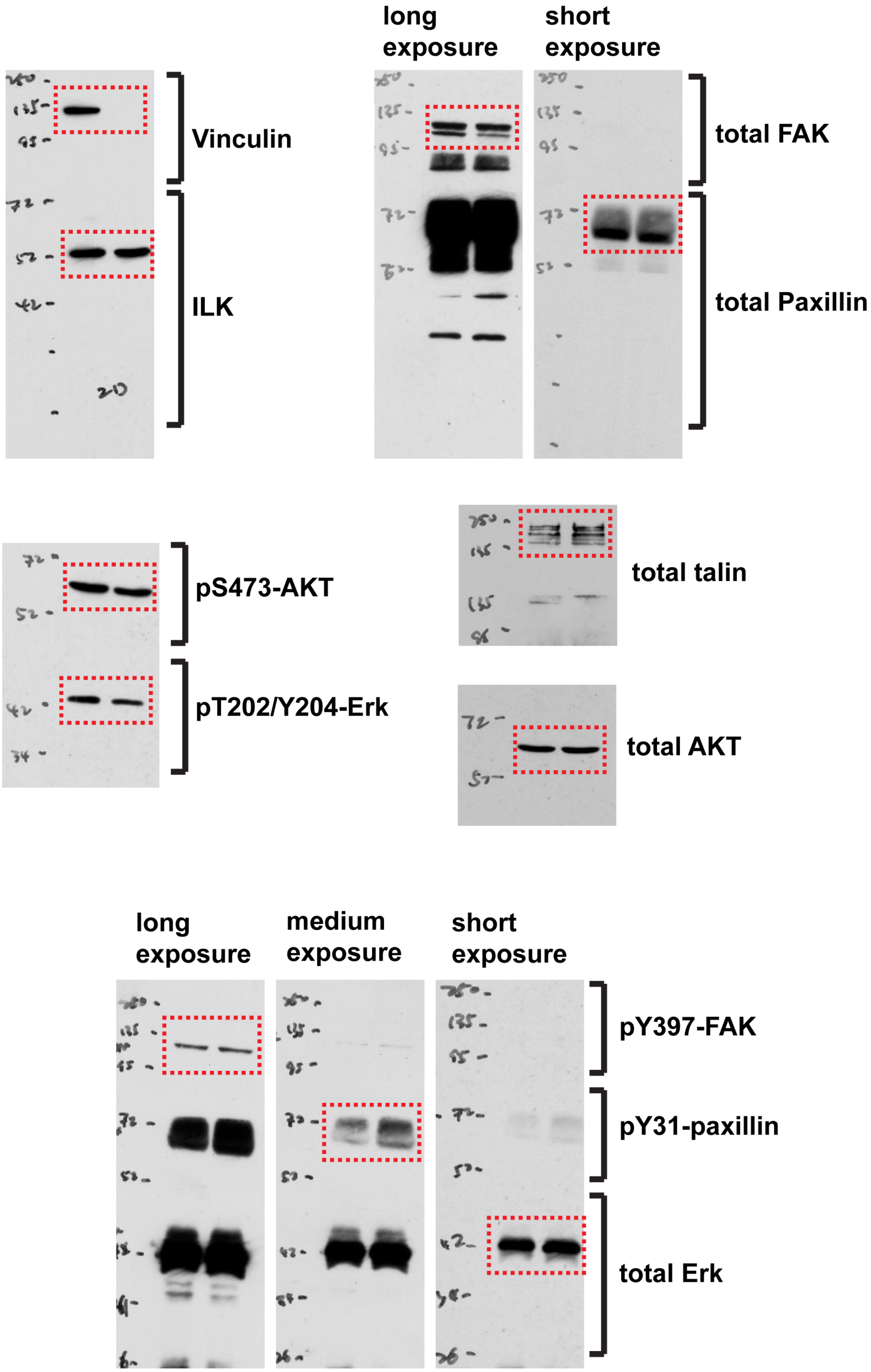

Uncropped blots Figure 2

Wang et al. Figure S5

Figure 4C

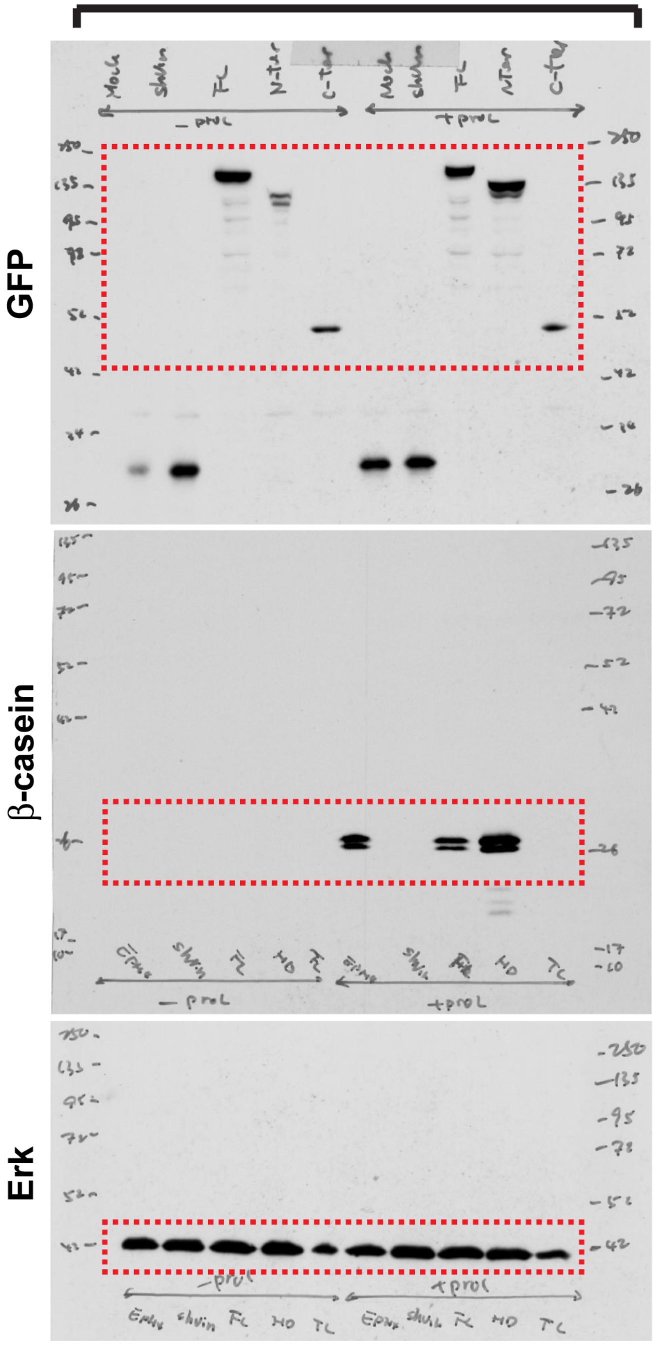

Figure 4G

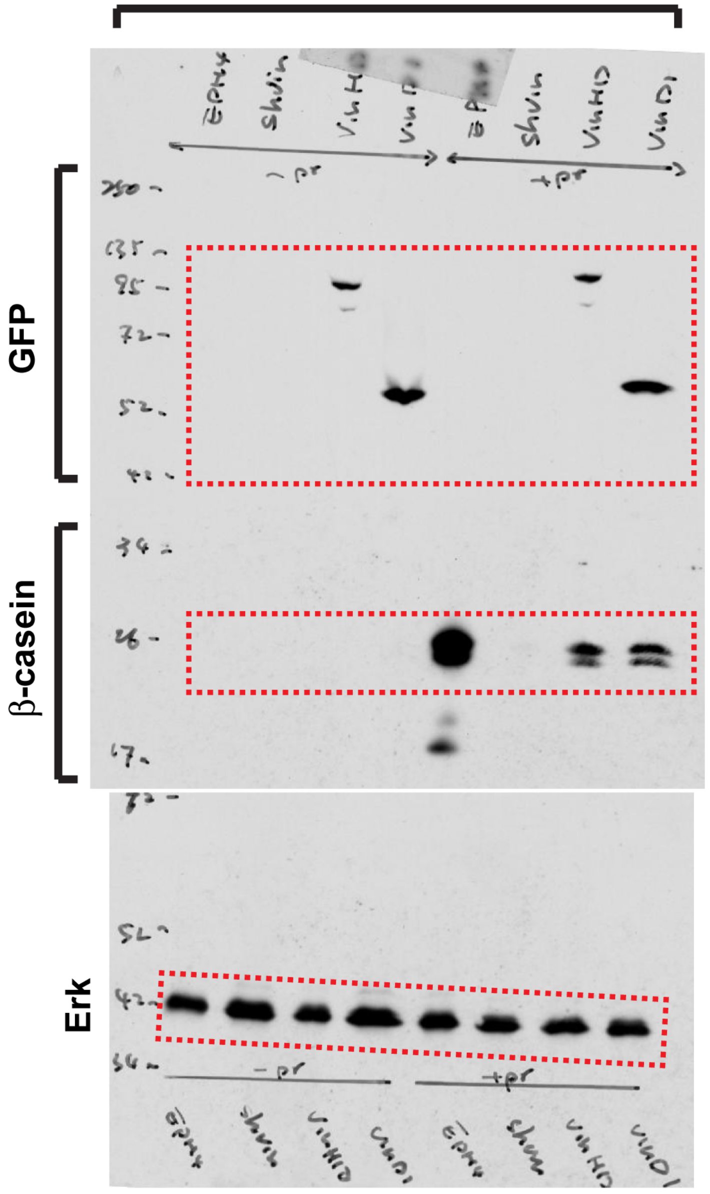

Uncropped blots Figure 4

Figure S3A

vinculin

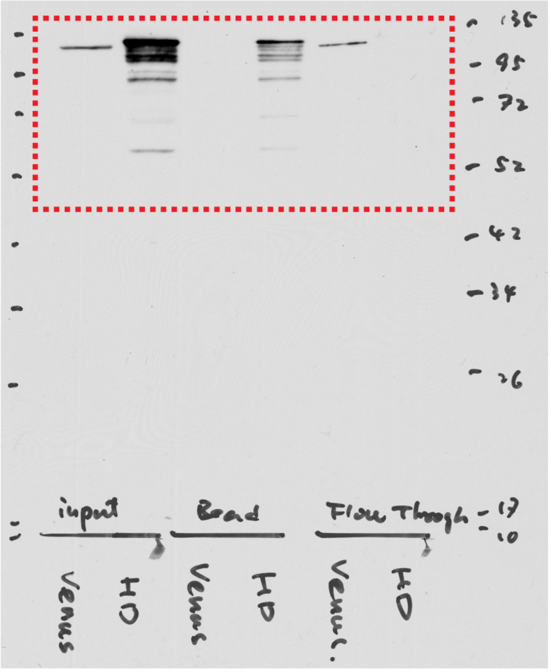

Erk

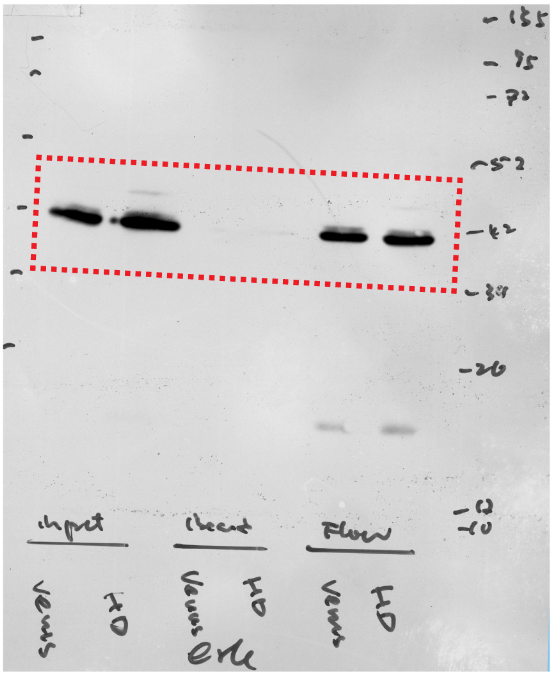

Figure S3B

vinculin

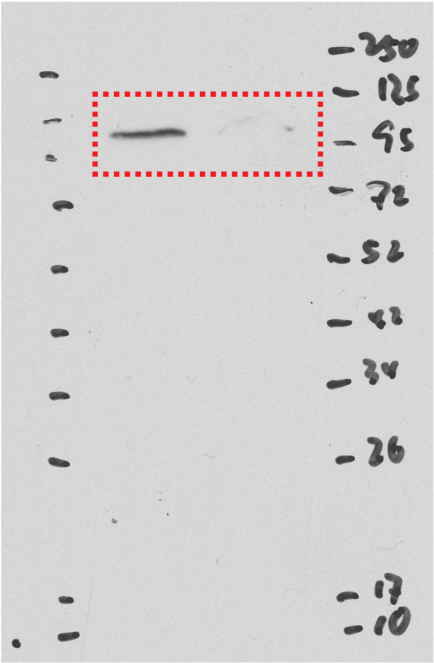

GFP

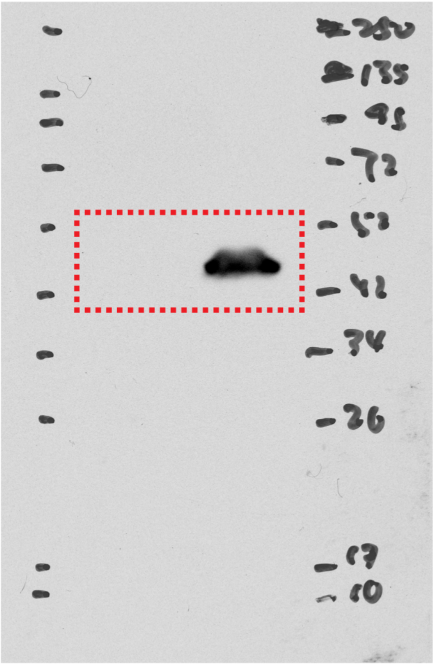

Erk

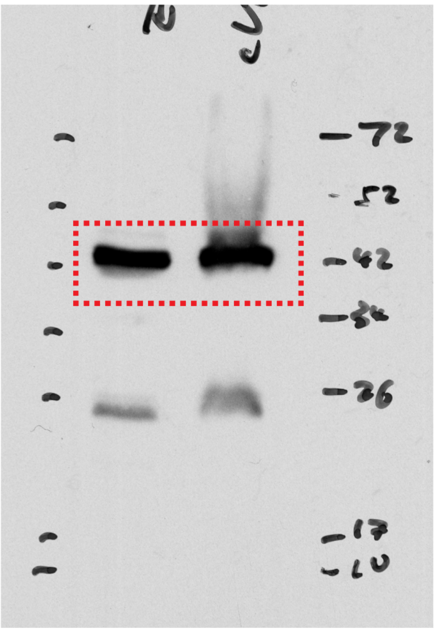

Uncropped blots Figure S3
